# Signaling lipids as diagnostic biomarkers for ocular surface cicatrizing conjunctivitis

**DOI:** 10.1007/s00109-020-01907-w

**Published:** 2020-04-20

**Authors:** Antonio Di Zazzo, Wei Yang, Marco Coassin, Alessandra Micera, Marco Antonini, Fabrizio Piccinni, Maria De Piano, Isabelle Kohler, Amy C. Harms, Thomas Hankemeier, Stefano Boinini, Alireza Mashaghi

**Affiliations:** 1 Ophthalmology Complex Operative Unit, University Campus Bio-Medico, Rome, Italy; 2 Leiden Academic Centre for Drug Research, Faculty of Science, Leiden University, 2333 CC Leiden, The Netherlands; 3 Research laboratories in Ophthalmology, IRCCS Fondazione Bietti, Rome, Italy; 4 Amsterdam Institute of Molecular and Life Sciences, Vrije Universiteit Amsterdam, Amsterdam, The Netherlands

**Keywords:** Mucous membrane pemphigoid, Cicatrizing conjunctivitis, Signaling lipid mediators, Metabolomics, LC-MS/MS

## Abstract

**Abstract:**

Metabolomics has been applied to diagnose diseases, predict disease progression, and design therapeutic strategies in various areas of medicine. However, it remains to be applied to the ocular surface diseases, where biological samples are often of limited quantities. We successfully performed proof-of-concept metabolomics assessment of volume-limited cytology samples from a clinical form of chronic inflammatory cicatrizing conjunctivitis, i.e., ocular MMP and discovered metabolic changes of signaling lipid mediators upon disease onset and progression. The metabolomics assessment revealed active oxylipins, lysophospholipids, fatty acids, and endocannabinoids alterations, from which potential biomarkers linked to inflammatory processes were identified. Possible underlying mechanisms such as dysregulated enzyme activities (e.g., lipoxygenases, cytochrome P450, and phospholipases) were suggested which may be considered as potential therapeutic targets in future studies.

**Key messages:**

- Metabolic profile of the ocular surface can be measured using impression cytology samples.
- Metabolomics analysis of ocular pemphigoid is presented for the first time.
- The metabolomics assessment of OCP patients revealed active oxylipins, lysophospholipids, fatty acids, and endocannabinoids alterations.
- Several oxylipins are identified as diagnostic biomarkers for OCP.

## Introduction

Chronic cicatrizing conjunctivitis (CCC) include 35 immunological and neoplastic entities which lead to ocular surface scarring, often causing ocular surface dysfunction and loss of visual ability [1]. Ocular mucous membrane pemphigoid (MMP) is the most common form of CCC and the high risk among the causes of blindness in western countries [2]. MMP is an autoimmune chronic inflammatory mucous disease characterized by subepithelial blistering, with an estimated incidence between 1:12000 and 1:60000 [3]. The ocular surface has the second highest approximate frequency of involvement, namely, circa 70% of cases [4]. A substantial proportion of cases, i.e., 27%, has ocular-only disease without any other mucous involvement [5]. The late diagnosis, which typically occurs once severe symptoms arise and only in advanced stages of fibrotic disease, limits the use of adequate treatments and positive outcomes [6, 7]. According to the MMP consensus conference in 2002 [4], the diagnostic confirmation of clinical suspicion requires direct immunofluorescence (DIF) on mucosal biopsies, demonstrating linear deposit of IgG/IgM/IgA/Complement fraction 3 at basement membrane. However, this diagnostic procedure shows low sensitivity, i.e., approximately 50–70% regardless of the site of biopsy [8, 9], leading to frequent false-negative results [5]. The necessity of DIF positivity for diagnostic confirmation is controversial but patients have similar outcome measures when the DIF is negative; furthermore, patients with ocular-only disease tend to have more negative results [5, 10].

This lack of diagnostic sensitivity critically affects a prompt and efficient medical intervention. Furthermore, a more objective method to identify both inflammatory and scarring activities to follow up the effects of therapy and ultimately guide treatment protocols remains strongly needed [1].

Immunometabolism is an emerging field in biomedicine that explores the crosstalk between inflammatory processes and metabolic signaling. Metabolic profiling is increasingly used to find novel disease biomarkers that inform on disease activity and therapeutic outcomes [11, 12], and provide insights into phenotypical variations between individual cells [13, 14]. The importance of metabolic and immunometabolic processes in ocular surface diseases has been increasingly reported [15–17]. In OCP patients, levels of tear matrix metalloproteinases (MMPs) and myeloperoxidase (MPO) are proved significantly elevated, and tissue inhibitor of metalloproteinase-1 (TIMP-1) decreased [18]; the novel technique of LC–MS/MS has been applied to detect cortisol to cortisone ratio in human ocular bio-fluids [19], and ocular surface impression cytology was used to determine the role of neutrophils as a biomarker of disease activity and progression [20]. Considering the inflammatory nature of ocular MMP, it is reasonable to assume that inflammatory metabolites may be actively involved, which justifies the use of a metabolomics-based approach to elucidate their roles in the onset and progression of ocular MMP. More specifically, signaling lipid mediators are bioactive lipids and lipid derivatives that mediate a variety of biological signaling events including pro-inflammatory, anti-inflammatory, or pro-resolving [21–23]. Metabolomics-based approaches targeting signaling lipid mediators are expected to significantly contribute to revealing inflammatory processes related to ocular MMP as well as potential disease biomarkers.

In the present study, we investigated whether a targeted metabolomics-based approach targeting signaling lipid mediators harvested by non-invasive conjunctival impression cytology could serve as sensitive and objective diagnostic biomarkers of active chronic inflammation which leads to ocular surface fibrosis in ocular MMP.

## Materials and methods

### Patients recruitment

We conducted an observational case-control pilot study of all new consecutive patients with diagnosis of ocular MMP who visited the Cornea service (Rome, Italy) from October to December 2017. Patients undergoing conjunctival biopsies older than 18 years with clinical signs of ocular MMP and who showed lymph-monocyte inflammatory cells at conjunctival histology were included in the study.

Enrolled subjects were further classified according to the Tauber-Foster clinical staging system [24], i.e., stage I, chronic conjunctivitis with subepithelial fibrosis; stage II, fornix restriction; stage III, symblepharon; and stage IV, ankyloblepharon. Stages II and III were subsequently grouped in four levels according scarring severity: a (< 25%), b (25–50%), c (50–75%), and d (> 75%). Patients with other ocular and/or associated systemic diseases or with modification of actual treatment within 3 months from the screening visit were excluded. Age- and sex-matched healthy volunteers, older than 18 years, with any family or personal history of ocular surface disease were recruited as control group.

All subjects received complete ocular clinical assessment with total symptom score (TSyS 0–24) and total sign score (TSS 0–30) calculated by the sum of each symptom and sign of the disease. Validated Ocular Surface Disease Index (OSDI) questionnaires were then used [25] (Table 1).

**Table 1:** Demographic and clinical characteristic of ocular MMP patients

Case	Age	Sex	Biopsy	T&F R	T&FL	Tsys R	Tsys L	TSS R	TSS L	OSDI
1	71	F	Positive*	IIB	IIB	15	13	8	6	40
2	48	F	Positive	IIB IIIA	IIB IIIA	9	27	5	5	70
3	65	F	Positive	IIC	IIB	14	14	12	10	68
4	65	M	Positive	I	I	13	12	6	4	40
5	34	M	Positive	IIA	IIB	7	7	7	6	40
6	68	F	Positive	IIB IIIB	IIB IIIB	7	10	12	12	69
7	82	M	Positive	IIC IIIC	IIB IIIC	11	13	8	7	50
8	74	F	Positive	IIB IIIC	IIB IIIB	12	11	8	4	25

*T&F*, Tauber and Foster classification; *R*, right eye; *L*, left eye; *Tsys*, total symptoms score; *TSS*, total sign score; *OSDI*, Ocular Surface Disease Index questionnaire; *T.T.*, topical therapy; *Positive*: lymphoplasmacellular infiltrates; Positive*, lymphoplasmacellular infiltrates and DIF positivity

All experimental procedures used in this study adhered to the tenets of the declaration of Helsinki concerning human subjects. The procedures performed were reviewed and approved by the Intramural Ethical Committee (University Campus Bio-Medico, Rome, Italy). All participants provided written informed consent for clinical and laboratory analysis.

### Impression cytology samples collection, shipment, and storage

After topical anesthesia with 0.04% oxybropocaine eye drops, two imprints (impression cytology, ICs) from each bulbar conjunctiva, specifically the inferior and temporal conjunctiva, were sampled using a Millipore membrane filter (0.22-μm membrane, Millipore, Milan, Italy). ICs were readily fixed by using a spray citofix (Bio-Fix Spray, Bio-Optica, Milan, Italy) according to a standardized procedure [26]. Conjunctival specimens of 32 Citofix samples were stored at − 80 °C until further analysis.

### Conjunctival biopsies and direct immunofluorescence

Conjunctival biopsies were obtained from clinically suspicious ocular MMP patients. Each biopsy conjunctival fragment was included in paraffin and sectioned to provide 5-μm sections for light/confocal microscopy. The DIF analysis was performed to identify the presence of a linear immunoglobulin deposition alongside the basement membrane zone (BMZ), according to the specific immunoreactivity (FC-coupled IgGAM antibodies, OBT0119F, Oxford Biotech., Oxford, UK), univocally present in ocular cicatricial pemphigoid positive sections. The basal histology included Giemsa (48900, Fluka, Milan, Italy), hematoxylin and eosin (HE, 05-M06014/05-M10002, Bio-Optica, Milan, Italy), and the periodic acid Schiff (PAS, 04-130802/05-M06002, Bio-Optica, Milan, Italy) stainings.

### Metabolic profiling using ultra-high performance liquid chromatography–mass spectrometry

The Citofix paper filters of collected samples were cut with a sterile scalpel and added to 200 μL of an ice-cold mixture composed of 80% methanol in water (v/v) to lyse cells, which were subsequently fixed on the filter prior to ultra-high performance liquid chromatography–mass spectrometry (UHPLC-MS/MS) analysis.

The sample preparation and UHPLC-MS/MS analysis were performed according to a validated profiling analytical platform [27, 28] with minor optimization. Briefly, samples were prepared using liquid-liquid extraction with a mixture of BuOH:MTBE (1:1, v/v). After evaporation of the supernatant, the dry residues were reconstituted in a mixture of MeOH:ACN (7:3, v/v) prior to UHPLC-MS/MS analysis. The developed and validated UHPLC-MS/MS method targeting different metabolic classes includes oxylipins, oxidative stress markers, endocannabinoids, and bile acids. Specifically, target metabolites include lysophospholipids (LPLs) such as lysophosphatidic acids (LPAs), lysophosphatidylethanolamines (LPEs), lysophosphatidylglycerols (LPGs), lysophosphatidylinositols (LPIs), and lysophosphatidylserines (LPSs); lipids from the sphinganine and sphingosine classes and their phosphorylated form; free fatty acids with a carbon chain length from C14 to C22; various isoprostane classes together with their respective prostaglandin isomers from different polyunsaturated fatty acids; oxylipins such as leukotrienes, thromboxanes, and hydroxyeicosatetraenoic acids; and endocannabinoids such as anandamide (AEA) and 1-arachidonoyl glycerol/2-arachidonoyl glycerol (1-AG/2-AG). The signaling lipid mediators were analyzed at two different mobile phase pH, namely, fatty acids, LPLs, sphingolipids, and taurine-conjugated bile acids at high pH and remaining metabolites at low pH, respectively. Low pH analyses were performed by a Shimadzu LCMS-8060 system (Shimadzu, Japan) using an Acquity BEH C18 column (50 × 2.1 mm, 1.7 μm; Waters, USA). The mobile phases were composed of water and 0.1% acetic acid (A); a mixture of ACN–MeOH (9:1, v/v) and 0.1% acetic acid (B); and IPA with 0.1% acetic acid (C). Separations were performed at 40 °C at a flow rate of 0.7 mL/min using the following gradient: 20% B and 1% C as starting conditions; changing to 85% B between 0.75 and 14 min and to 15% C between 11 and 14 min; conditions held for 0.5 min prior to column re-equilibration at the starting conditions from 14.8 to 16 min. High pH analyses were performed by a Shimadzu LCMS-8050 system (Shimadzu, Japan) using a Kinetex® Core-Shell EVO 100 Å C18 column (50 × 2.1 mm, 1.8 μm; Phemomenex, USA). The mobile phases consisted of aqueous phase A (95% water and 5% ACN with 2 mM ammonium acetate and 0.1% ammonium hydroxide) and organic phase B (95% ACN and 5% water with 2 mM ammonium acetate and 0.1% ammonium hydroxide). Separations were performed at 40 °C at a flow rate of 0.6 mL/min and with the following gradient: 1% B as starting conditions; increasing to 100% B from 0.7 to 7.7 min; 100% B hold for 0.75 min prior to re-equilibration at the starting conditions between 8.75 and 11 min. MS/MS acquisition was performed in both electrospray ionization positive and negative mode using multiple reaction monitoring (MRM) with polarity switching.

### Data pre-processing

Peak integration was performed using LabSolutions (Shimadzu, Version 5.65). For all metabolites, the peak area was corrected using internal standards, by calculating the ratio of peak area of the target compound to the peak area of the assigned internal standard to obtain relative quantitation for each metabolite. Acquired peak area ratios of each metabolite were performed with statistical analysis using IBM SPSS Statistics (Version 25.0) and GraphPad Prism (Version 7.0).

### Data analysis

All patients’ scores for all outcome variables were calculated for both eyes, with 95% confidence interval for each outcome measure. The differences between mean values for the different time points were assessed with one-way ANOVA nonparametric test, and the differences between mean values between control and patient groups were assessed with independent nonparametric test. A *p* value of 0.05 was chosen as the limit for statistical significance.

Comparative analyses were performed using peak area ratios of each metabolite to (1) stratify metabolic differences between controls and ocular MMP patients, (2) identify potential signaling lipid mediators as diagnostic biomarker candidate, and (3) analyze metabolite dynamics associated with ocular MMP progression. For all comparative analyses, a *p* value of 0.05 was selected as cut-off value for statistical significance.

## Results

### Observational case-control study

**Fig. 1:**
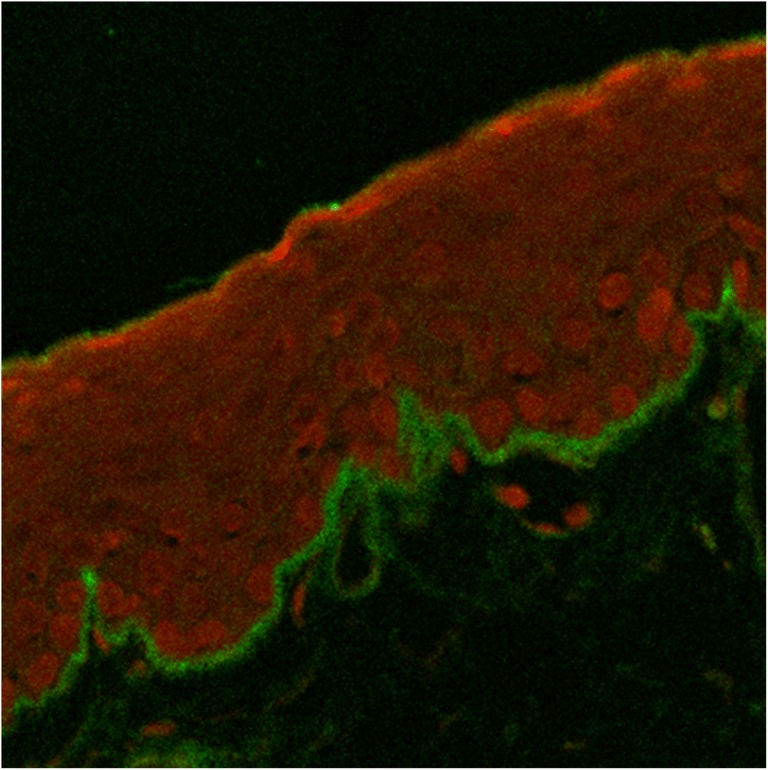
Confocal analysis of ocular cicatricial pemphigoid diagnosis by polyvalent IgG. Direct immune fluorescence IgG/propidium iodide, merge, × 20

Eight patients with clinical manifestation and histological diagnosis of ocular MMP (34–82 years; mean age 63.4; M/F = 1) were enrolled in an observational case-control pilot study. Eight healthy volunteers (40–88 years; mean age 71; M/F = 1), undergoing routine age-related cataract surgery, were included in the control group. Table 1 lists the clinical features and demographics of included patients. Only 12.5% of recruited patients showed positive DIF after conjunctival biopsy (Fig. 1).

### Targeted metabolomics of controls and ocular MMP patients

**Fig. 2:**
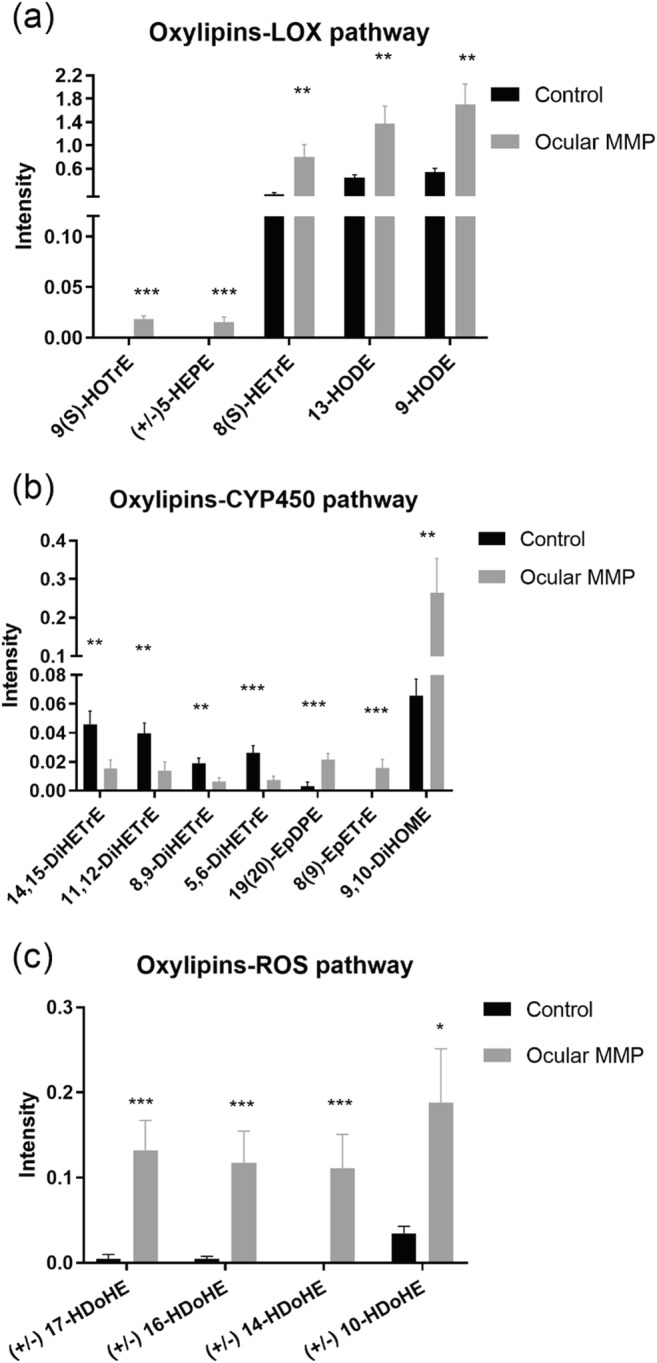
Signaling lipid mediators (oxylipins) in control group and ocular MMP patients. **a** Oxylipins-LOX pathway. **b** Oxylipins-CYP450 pathway. **c** Oxylipins-ROS pathway. The intensity represents the relative quantitation of each metabolite

Numerous significant differences in the targeted signaling lipid mediators assessed using UHPLC-MS/MS in conjunctival biopsies were observed in MMP patients compared with the control group. Figures 2 (oxylipins), 3 (lysophospholipids and fatty acids), and 4 (endocannabinoids) illustrate the metabolite differences observed between the two groups. Overall, 16 oxylipins (i.e., five metabolites derived from the lipoxygenase [LOX] pathway, seven derived from the CYP450 pathway, and four metabolites associated to oxidative stress), six lysophosphatidic acids (i.e., three LPAs and three cyclic LPAs [cLPAs]), 10 lysophosphatidyl-derived lipids (i.e., three LPEs, two LPGs, two LPIs, and three LPSs), seven free fatty acids, and 10 endocannabinoids showed significantly altered levels between healthy controls and ocular MMP patients. To be specific, oxylipins showed accumulated levels in conjunctival biopsies of ocular MMP patients compared with healthy controls. Moreover, LPA (18:0), cLPA (16:1), cLPA (18:0), cLPA (18:1), LPE (16:0), LPE (18:0), LPG (16:0), LPG (18:0), LPI (16:0), LPI (18:0), LPS (16:0), LPS (18:0), and LPS (18:1) were detected at significantly reduced levels in the ocular MMP patient group while LPA (20:3), LPA (22:6), and LPE (22:4) were found at significantly higher levels in the ocular MMP group. In the free fatty acid class, only oleic acid (18:1, ω-9) was more abundant in the patient group while the other targeted polyunsaturated fatty acids were all detected at lower levels in the patient group. Finally, the detected endocannabinoids showed lowered levels in ocular MMP patients, except for 2-arachidonyl glyceryl ether (2-AGE) which was more abundant in the patient group.

**Fig. 3:**
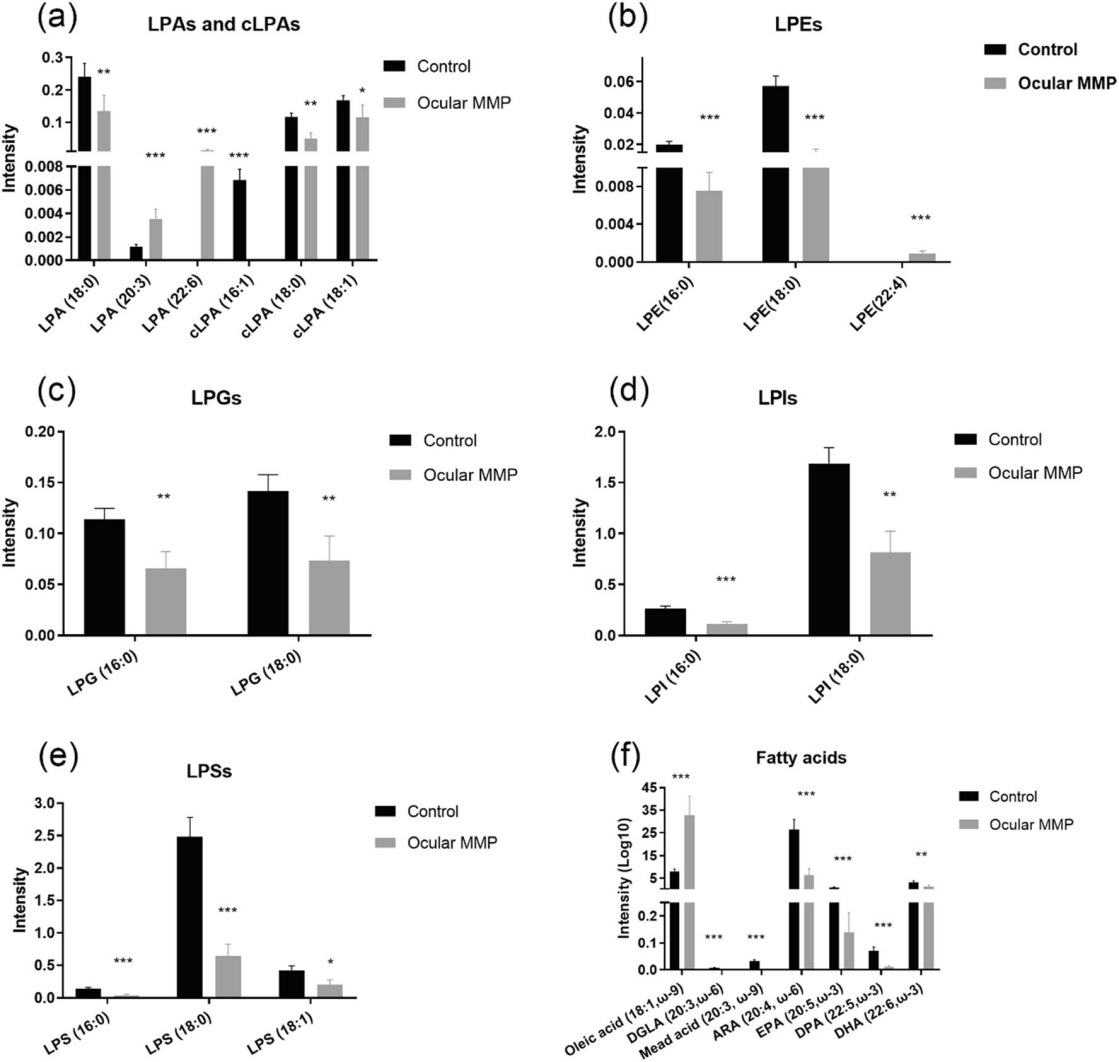
Signaling lipid mediators (lysophospholipids and fatty acids) in control group and ocular MMP patients. **a** Lysophosphatidic acids (LPAs) and cyclic-lysophosphatidic acids (cLPAs). **b** Lysophosphatidylethanolamines (LPEs). **c** Lysophosphatidylglycerols (LPGs). **d** Lysophosphatidylinositol (LPIs). **e** Lysophosphatidylserines (LPSs). **f** Fatty acids. The intensity represents the relative quantitation of each metabolite

**Fig. 4:**
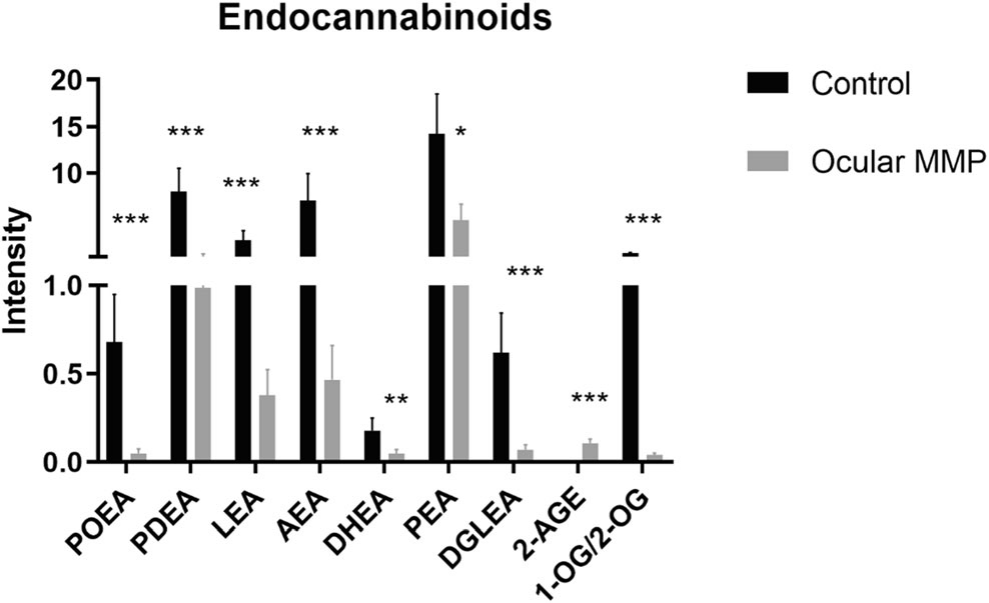
Signaling lipid mediators (endocannabinoids) in control group and ocular MMP patients. The intensity represents the relative quantitation of each metabolite

### Signaling lipid mediators as potential biomarker candidates for ocular MMP diagnosis

**Fig. 5:**
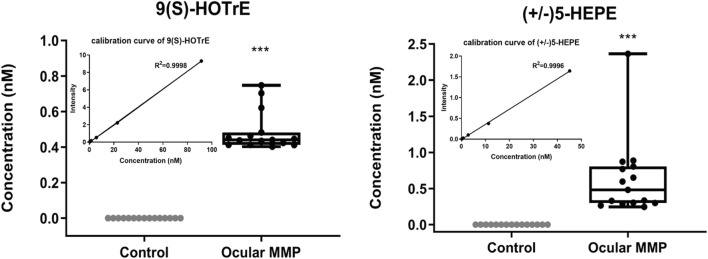
Signaling lipid mediators as potential biomarker candidates for ocular MMP diagnosis: 9(S)-HOTrE and (±)5-HEPE. Data is shown in absolute concentration (nM) of the metabolite in conjunctival biopsies in controls and ocular MMP patients, respectively, calculated based on the calibration curve which is shown in the insert figure

Among the signaling lipid mediators detected, two metabolites, i.e., 9(*S*)-HOTrE and (± )5-HEPE, may be suggested as potential biomarker candidates for ocular MMP diagnosis. Indeed, those two metabolites were exclusively detected, and at considerable abundance, in all patients’ conjunctival biopsies analyzed, as illustrated in Fig. 5, while not detected in control conjunctival biopsies. This suggests that 9(*S*)-HOTrE and (±)5-HEPE were both only produced in ocular MMP patients, which makes them potential specific disease biomarker candidates.

### Signaling lipid mediators as ocular MMP staging indicators

**Fig. 6:**
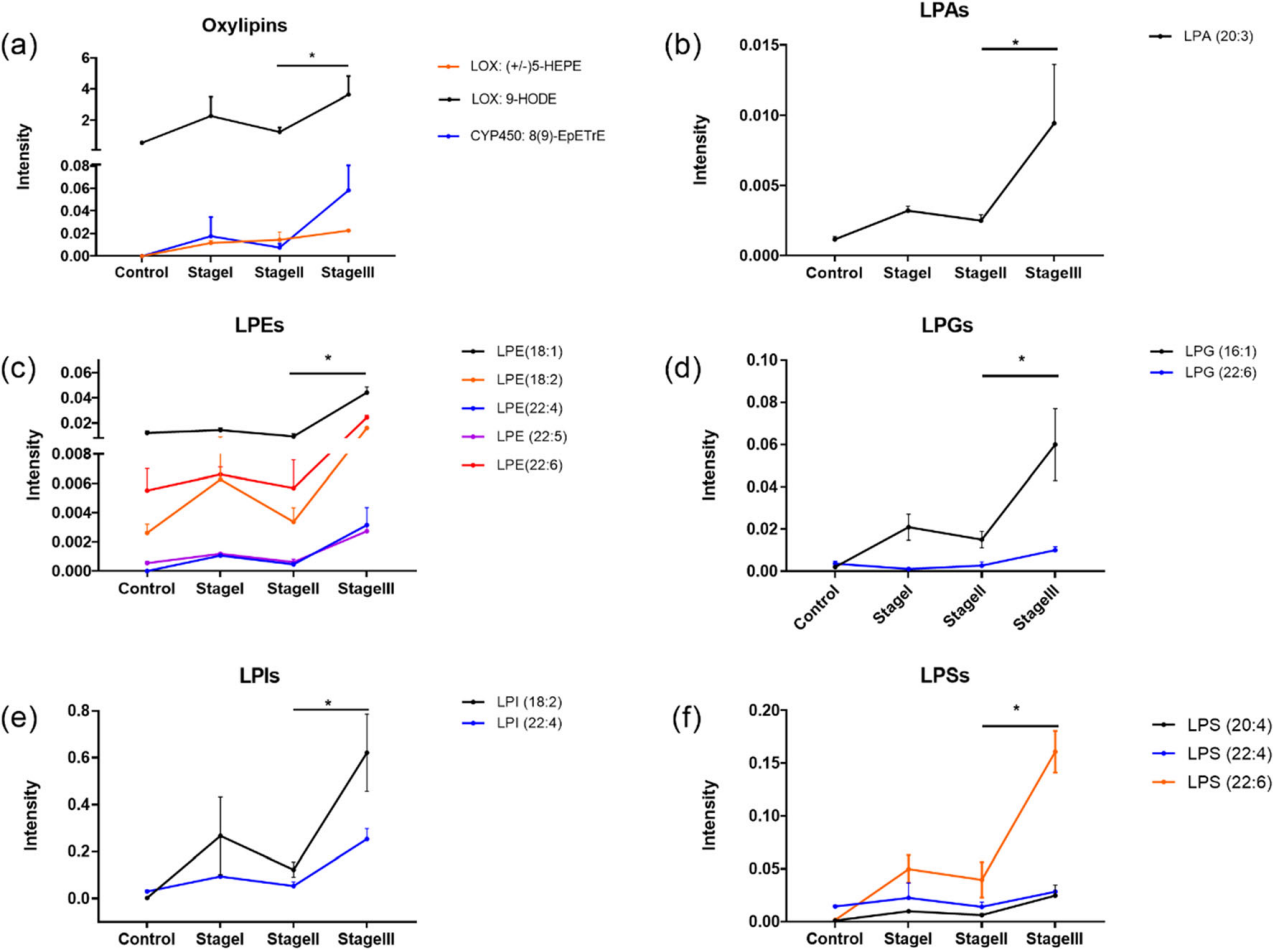
Signaling lipid mediators as mid-late-staging indicators. A panel of oxylipins (**a**) and lysophospholipids (**b**–**f**) significantly increased from stage II to stage III. The intensity represents the relative quantitation of each metabolite

The comparative analyses performed among ocular MMP patients highlighted the metabolic dynamic changes associated with disease progression. Figure 6 shows that the signaling lipid mediators were significantly elevated in ocular MMP stage III. Specifically, (±)5-HEPE, 9-HODE, 8(9)-EpETrE, LPA (20:3), LPE (18:1), LPE (18:2), LPE (22:4), LPE (22:5), LPE (22:6), LPG (16:1), LPG (22:6), LPI (18:2), LPI (22:4), LPS (20:4), LPS (22:4), and LPS (22:6) all showed elevated levels in the stage III compared with stage II ocular MMP samples. The results showed metabolic changes in conjunctival cells, which have associations with deteriorative characteristics at the advanced stage.

## Discussion

Ocular MMP is a complex condition easily misdiagnosed in first stages when it appears as a recurrent chronic conjunctivitis. More than 80% of patients are reported to have a stage III Tauber-Foster or higher at presentation [6, 7]. The symptoms can affect quality of life of patients, years before reaching an accurate diagnosis and thus, the appropriate systemic immunomodulatory therapy (IMT). DIF is the gold standard diagnostic test but shows a low diagnostic sensitivity, particularly in MMP with ocular manifestation alone. Even if IMT is a successful approach for controlling the clinically significant ocular surface inflammation, the subclinical fibrotic process continues in 40–50% of patients [7, 24]. Moreover, little is known about the relative effectiveness of IMT drugs to slow down or stop the fibrosis. The disease progression is also difficult to assess due to the intrinsic limits of the Tauber-Foster staging system. Recently, fibroblasts from MMP patients’ conjunctiva have shown to have a pro-fibrotic phenotype compared with a healthy population, preserved when isolated in vitro [29]. Our study aimed to show that metabolomics-based analysis of ICs samples is a rapid and non-invasive approach for identification of novel diagnostic biomarker candidates as well as new potential therapeutic targets. Indeed, metabolomics could define the biochemical status of each sample and the differential expression in signaling lipid mediators, giving to clinicians new opportunities in diagnosis, prognosis, and staging of the disease.

Previously reported evidence suggested the roles of various signaling lipid mediators in (patho)physiological processes. For example, lysophospholipids (LPLs), which are minor structural components of biological membranes, have been already reported to be important signaling mediators of a wide range of biological effects as well as markers of specific biological processes [30–32]. Indeed, LPEs and LPGs are involved in physiological extracellular matrix remodeling [33] as well as in chronic inflammatory fibrotic disease, such as idiopathic pulmonary fibrosis, by inducing TGF-β release and activation [34]. Another example is prostaglandin F_2α_ (PGF_2α_), which is specifically elevated during acute inflammatory response in several tissues such as airways, uterus, vessels’ smooth muscle cells, and eye [35].

In this study, a metabolomics-based approach targeting signaling lipid mediators showed a great potential in revealing immunometabolic changes associated with ocular MMP onset and progression. As illustrated in Fig. 2, the oxylipins associated with elevated levels in ocular MMP samples were mostly issued either from enzymatic pathways such as LOX and CYP450 or by non-enzymatic auto-oxidation. Oxylipins are oxidized lipid mediators derived from fatty acids with various carbon number and double bonds [36]. Omega-3 fatty acids such as eicosapentaenoic acid (EPA, 20:5) and docosahexaenoic acid (DHA, 22:6) and omega-6 fatty acids such as arachidonic acid (ARA,20:4), linoleic acid (LA, 18:2), and dihomo-γ-linolenic acid (DGLA, 20:3) have been reported with anti-inflammatory or pro-inflammatory properties [37, 38]. As supported by Figs. 2a and 3f, increased levels of (±)5-HEPE were detected in the patient group while its precursor EPA was found at significantly lower levels. In addition, 8(S)-HETrE was more abundant in the patient group while the concentration of its upstream fatty acid DGLA accordingly decreased. A similar trend was observed in oxylipins issued from the CYP450 pathway, as shown in Fig. 2b, where 19(20)-EpDPE was present at higher concentrations in patient samples while its precursor fatty acid DHA was reduced. Interestingly, the DiHETrE series of lipid mediators (i.e., 14,15-DiHETrE, 11,12-DiHETrE, 8,9-DiHETrE, and 5,6-DiHETrE) derived from ARA were less converted through CYP450 pathway, which may be explained by a shift towards the more active production of 8,9-EpETrE from the same precursor ARA. This is supported by the fact that the DiHETrE series of oxylipins are downstream products of the EpETrE series, a conversion that might be less active in the patient group compared with controls. Figure 2c shows five oxylipins of the HDoHE subclass which were present at significantly higher levels in conjunctival biopsies of ocular MMP patients, suggesting a probable overactive peroxidation of DHA to HDoHEs due to excess of reactive oxygen species (ROS). The occurrence of tissue fibrosis has been suggested to correlate with the increase of ROS-related oxidative stress [39, 40]. Therefore, we hypothesize that overproduction of HDoHEs, which may be considered an oxidative stress readout, may facilitate conjunctivitis and subepithelial fibrosis.

From a clinical diagnosis perspective, the detected metabolites with exclusive expression in ocular MMP compared with the control group show a promising role in assisting diagnosis. For instance, two oxylipins 9(*S*)-HOTrE and (±) 5-HEPE were exclusively detected in the patient group with an average concentration level ~ 0.5 nM in conjunctival biopsies. Other potential diagnostic markers could be LPE (22:4) and LPA (22:6) which were exclusively expressed in the patient group as well. However, for the latter compounds, no absolute concentration could be assessed due to the lack of commercially available reference standards.

The oxylipins (±)5-HEPE, 9-HODE, and 8(9)-EpETrE all featured an accumulating trend from stage II to stage III where the symblepharon appears, being potential indicators underlying the metabolic dynamics associated with ocular MMP progression. (±)5-HEPE and 9-HODE are 5-LOX-mediated lipid mediators; a pathological activity of 5-LOX may explain their abnormal surplus associated with MMP staging. This hypothesis is supported by the evidence of age-dependent increase of 5-LOX and oxidative stress [41, 42] as well as excess formation of cytotoxic mediators due to overactivation of 5-LOX [43, 44].

LPLs also showed differential concentrations in the patient versus control groups. Of interest is the observation that saturated and mono-unsaturated LPLs were detected at lower concentrations while the polyunsaturated compounds were remarkably higher in the ocular MMP group. LPLs are breakdown products of membrane phospholipids, a phenomenon that occurs by losing one of the fatty acid chains (sn-1 or sn-2) through the action of phospholipase, with a different cleavage position depending of the phospholipase. Generally, sn-1 chain is saturated or mono-unsaturated while sn-2 chain is usually polyunsaturated. Phospholipases A1 (PLA1s) remove fatty acyl groups from sn-1 position of phospholipids, producing saturated/mono-unsaturated fatty acids (oleic acid, palmitic acid, and stearic acid as common sn-1 chains) and polyunsaturated LPLs. On the other hand, if the cleavage happens at sn-2 position by phospholipases A2 (PLA2s), polyunsaturated fatty acid chains are generated (i.e., ARA, DHA, EPA as common sn-2 chains) together with saturated/mono-unsaturated lysophospholipids [45]. Therefore, the observed trends for the detected fatty acids, LPLs, and derived LPAs (Figs. 3 and 6) could result from hyperactive PLA1s and/or hypoactive PLA2s in ocular MMP patients and in patients at a different disease stage. Even though PLA2s have been reported to be overexpressed or overactive in some inflammatory conditions [45], they seem to have opposite roles in terms of ocular disease where PLA2s were found to be protective against pterygium formation, while downregulated expression of PLA2s has been reported in pterygium tissues compared with normal conjunctiva [46]. Further investigations are therefore needed to elucidate the enzymatic changes in ocular MMP.

The endocannabinoid system (ECS) is composed of cannabinoid receptors, biosynthetic, and degradative enzymes as well as endocannabinoids. The latter are lipid-based signaling molecules involved in regulation of physiological and cognitive processes including fertility, pregnancy, pain sensation, mood, and memory [47]. Particularly, the ability of modulating inflammation and pain brings endocannabinoids as potential therapeutic targets [48, 49]. In this study, a panel of endocannabinoids was detected at significantly lower levels in ocular MMP (Fig. 4), suggesting a protective role in physiological ocular environment. With gaining momentum in treating ocular diseases [47], endocannabinoids have been proposed as therapeutic approaches in glaucoma [50] and retinal disease [51]. However, the potential role of endocannabinoids in ocular MMP remains largely unexplored. Our research revealed for the first time the lower levels of endocannabinoids detected in ocular MMP compared with the control group. More interestingly, a remarkable decline of AEA, PEA, PDEA, and LEA was observed from ocular MMP stage I to stage III; all levels were also lower than in the control group (data now shown). This may suggest that these compounds could contribute to the onset of ocular MMP and the development of the disease. Altogether, endocannabinoids may protect against the onset and progression of ocular MMP, showing their potential for the development of novel therapeutic strategies for treating ocular MMP.

## Conclusions

The present study demonstrates for the first time the power of metabolomics in conjunctival impression cytology and reveals altered levels of signaling lipid mediators associated with ocular MMP onset and progression. Multiple metabolites from different classes, i.e., oxylipins, LPLs, fatty acids, and endocannabinoids, showed significantly different levels in conjunctival biopsies of ocular MMP patients compared with healthy controls, which suggests an underlying metabolic reprogramming occurring in ocular MMP. Probable mechanisms could be the involvement of inflammatory signaling lipid mediators abundantly produced through overactive LOX and cytochrome P450 enzymes, or through non-enzymatic lipid peroxidation, as well as disruption of PLA1s and PLA2s. On the other hand, endocannabinoid deficiency may promote the disease onset and development. The specific presence of 9(S)-HOTrE and (±)5-HEPE in the ocular surface in MMP patients can be exploited as potential diagnostic biomarker candidates. Accumulating oxylipins and LPLs at mid-late stage of ocular MMP indicate dynamic variations of conjunctival cell metabolism with aggravating inflammation underlying the disease progression. Moreover, these findings could offer better treatment options and provide potential therapeutic targets for future interventions.

Limitations in this study include small sample size and the lack of further cicatrizing conjunctivitis patients as controls. Future perspectives include the validation of these findings in a larger population of ocular MMP patients and patients affected with other challenging eye surface conditions as primary Sjogren’s syndrome, a topical keratoconjunctivitis, and Steven-Johnson syndrome. Moreover, a larger selection of metabolites will be added to the developed UHPLC-MS/MS method to increase the validity of these results and offer a broader knowledge of the eye surface at the metabolite level.
